# Aeroallergens in Canada: Distribution, Public Health Impacts, and Opportunities for Prevention

**DOI:** 10.3390/ijerph15081577

**Published:** 2018-07-25

**Authors:** Cecilia Sierra-Heredia, Michelle North, Jeff Brook, Christina Daly, Anne K. Ellis, Dave Henderson, Sarah B. Henderson, Éric Lavigne, Tim K. Takaro

**Affiliations:** 1 Faculty of Health Sciences, Simon Fraser University, Burnaby, BC V5A 1S6, Canada; sierrahe@sfu.ca; 2 Institute of Medical Science, University of Toronto, Toronto, ON M5S 3H7, Canada; m.north@utoronto.ca; 3 Department of Biomedical & Molecular Sciences and Division of Allergy & Immunology, Department of Medicine, Queen’s University, Kingston, ON K7L 3N6, Canada; Anne.Ellis@kingstonhsc.ca; 4 Allergy Research Unit, Kingston General Hospital, Kingston, ON K7L 2V7, Canada; 5 Dalla Lana School of Public Health, University of Toronto, Toronto, ON M3H 5T4, Canada; jeff.brook@canada.ca; 6 Air Quality Health Index, Health Canada, Ottawa, ON K1A 0K9, Canada; christina.daly@canada.ca; 7 Health and Air Quality Services, Environment and Climate Change Canada, Gatineau, QC K1A 0H3, Canada; dave.henderson@canada.ca; 8 Environmental Health Services, BC Centre for Disease Control, Vancouver, BC V5Z 4R4, Canada; sarah.henderson@bccdc.ca; 9 School of Population and Public Health, University of British Columbia, Vancouver, BC V6T 1Z3, Canada; 10 Air Health Science Division, Health Canada, Ottawa, ON K1A 0K9, Canada; eric.lavigne@hc-sc.gc.ca; 11 School of Epidemiology and Public Health, University of Ottawa, Ottawa, ON K1G 5Z3, Canada

**Keywords:** aeroallergens, pollen, fungi, allergy, asthma, atopic march, Canada

## Abstract

Aeroallergens occur naturally in the environment and are widely dispersed across Canada, yet their public health implications are not well-understood. This review intends to provide a scientific and public health-oriented perspective on aeroallergens in Canada: their distribution, health impacts, and new developments including the effects of climate change and the potential role of aeroallergens in the development of allergies and asthma. The review also describes anthropogenic effects on plant distribution and diversity, and how aeroallergens interact with other environmental elements, such as air pollution and weather events. Increased understanding of the relationships between aeroallergens and health will enhance our ability to provide accurate information, improve preventive measures and provide timely treatments for affected populations.

## 1. Introduction

Over the past 70 years, the prevalence of allergic conditions has increased in high-income countries to the point of becoming a worldwide public health concern [1]. This increase cannot be explained by genetic changes only [2]. Environmental exposure to air pollutants and aeroallergens have been hypothesized as potential causes for this steep increase, as the nature of these exposures have changed during a similar window of time [3].

Environmental factors play a key role in defining the type of sensitization and the kind of atopic disease in genetically at-risk individuals. While heredity regulates the intergenerational transmission of the susceptibility to different phenotypes of atopic responsiveness, environmental exposure is a crucial factor in the development of atopic disease. The environmental exposures linked to the development of allergies are to: (a) common aeroallergens (e.g., pollen or mold spores); and (b) pollution, like particulate matter (PM) or environmental tobacco smoke (ETS), among others [4].

The environmental exposures listed produce a specific imbalance in the immune system [5]. There are two types of T helper immune cells (Th1 and Th2) that appear imbalanced in allergic conditions [6], with a higher proportion of the Th2 cell population [7]. The hygiene hypothesis is also considered as one of the reasons for the specific imbalance in the immune system [8]. This hypothesis attributes a reduced exposure to infectious agents at key times of immune system development to the Th2 predominance. This skewed microbial exposure may be due to improved hygiene and sanitation, and increased use of disinfectants and antibiotics [9]. When an individual has this type of imbalanced immune system, repeated exposures to a myriad of specific proteins (e.g., pollen, certain molds, dust mite feces, eggs, peanuts) can induce the creation of IgE antibodies [10]. These initial encounters with the proteins produce a sensitization to these specific molecules, and subsequent exposures will trigger symptoms each time [10,11].

Allergy symptoms in response to pollen and fungal spores are driven by IgE that is specific to proteins found in these spores [12]. The allergenicity of pollen spores refers to the concentration of protein epitopes in each spore, to which specific IgE molecules produced by the plasma cells of allergic individuals may bind [13]. Certain pollen grains are known to be highly allergenic (e.g., ragweed a.k.a *Ambrosia*), and highly prevalent in some parts of Canada [14]. This combination of allergenicity and prevalence makes some ambient aeroallergens highly problematic due to the growing number of exposed and sensitized individuals [15]. This exposure and the physical symptoms of the immune response have a direct impact on the everyday lives of patients with allergic conditions. They also have an impact at the societal level, including costs to the health systems that address medical needs associated with allergic responses.

This review will describe the current state of our knowledge about the variety and distribution of plant and fungi-derived aeroallergens in Canada. We will also discuss allergic diseases and the health impacts that Canadian allergy sufferers experience in connection with aeroallergens and the weather and climatic conditions that enhance exposure. It is our hope that this public health-oriented review will enhance the ability of researchers, health practitioners, and governments to provide accurate information to the public and improve preventive measures and treatments.

## 2. Review

### 2.1. Introduction to Ambient Aeroallergens

Pollen grains function as a container for the male gametophytes of plants [16]. The essential reproductive event among higher plants is achieved only when the pollen grain is successfully transferred from the floral anther to the recipient stigma. The strategies that plants use in their pollen transfer are classified into two categories: entomophilous plants depend on organisms such as insects or hummingbirds to carry larger, stickier pollen grains, anemophilous plants release large quantities of pollen grains to be blown in the wind [17].

**Figure 1:**
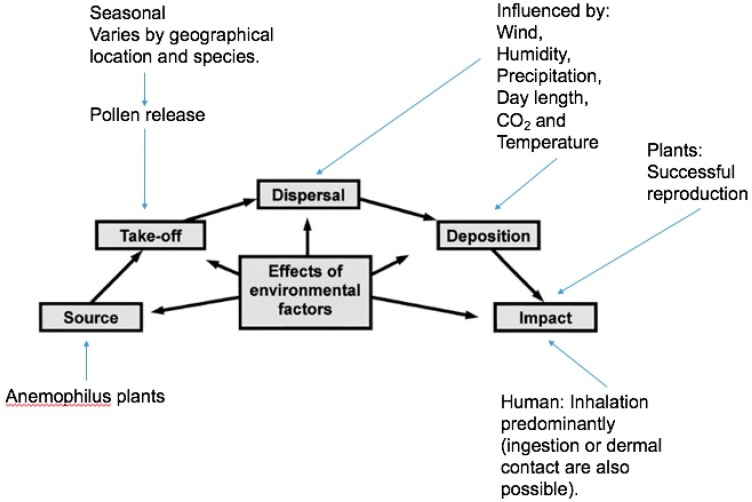
The aerobiology pathway of pollens, adapted from [21].

When anemophilous plants release pollen, a number of environmental factors affect how, when, and how many grains reach their final destination [18]. The aerobiology pathway of pollen (as seen on Figure 1) illustrates how pollen grains can travel from their source to interact with humans and potentially affect health. Many of these factors are affected by shifting climatic conditions [19,20].

Currently, more than 150 pollen allergens are curated by the International Union of Immunological Societies [22]. From the entire list of pollen allergens, 12 are of particular interest in Europe due to their abundance in the atmosphere [23] and allergenic potency [24] (p. 10): (1) ragweed (*Ambrosia*); (2) alder (*Alnus*); (3) mugwort (*Artemisia*); (4) birch (*Betula*); (5) goosefoots (*Chenopodiaceae*); (6) hazel (*Corylus*); (7) cypresses, including yews (*Cupressaceae/Taxaceae*); (8) olive (*Olea*); (9) plane tree (*Platanus*); (10) grass (*Poaceae*); (11) oak (*Quercus*); (12) and wall pellitory (*Urtica/Parietaria*).

In Canada, birch and grasses are more abundant than ragweed, which is found mostly in Ontario and Quebec. Cypress, olive, plane tree, and wall pellitory are not present in Canada [14]. 

### 2.2. Canadian Distribution of Plant-Derived Aeroallergens

**Figure 2:**
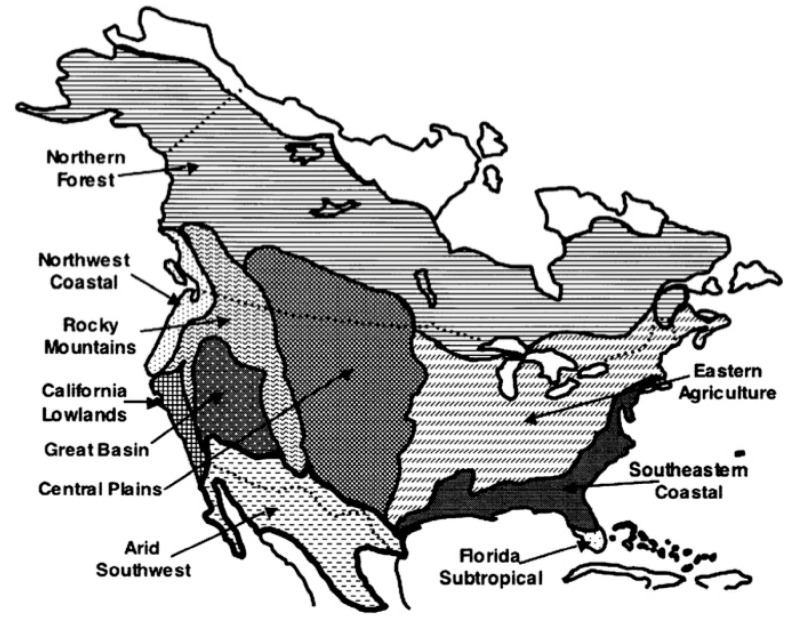
Floristic zones of North America, from [14].

The geographical distribution of species of plants is dependent on multiple factors, including precipitation, soil composition and moisture and average high and low temperatures [14]. Five “floristic zones” that support the growth of different aeroallergen-producing plants have been defined across Canada [14]. As seen on Figure 2, from west to east, they are: Northwest Coastal, Northern Forest, Rocky Mountain, Central Plains, and Eastern Agricultural [14]. The major allergenic plants that grow in each of the floristic zones are widely varied (Table S1 in the supplementary materials). 

Trees, grasses, and ragweed are the most common allergens associated with outdoor pollen-induced allergies across Canada [25,26,27]. Short ragweed is highly cross-reactive with all other ragweed species, as well as sage and mugwort [25,26,28,29,30]. Additionally, there is substantial clinical cross-reactivity between tree and grass allergenic species [27]. For example, individuals allergic to birch pollen in Northern Sweden have also been sensitized to beech pollen (*Fagus*), although that species does not grow in the region [31]. 

Local factors can affect spatial variation within a region [32]. Examples of the local factors are: tree canopy, defined as the types of trees planted in the geographical area of interest [33]; and level of urbanization, defined considering street network coverage and quantity of vegetation [34]. The amount of pollen in the air at a particular location depends on the distance and number of source plants in the area, atmospheric conditions and plant physiological factors such as the number of accumulated degree days at which their pollen is released [35]. Pollen grains can break open to release submicronic pollen-derived bioaerosols containing allergenic proteins, particularly when the grains have been aloft for some time and/or become wet [36,37,38,39] as in thunderstorm asthma. Some increased exposure to aeroallergens is also due to human activity, such as landscaping practices that create niches favoring the growth of weeds such as ragweed [35].

### 2.3. Fungal Aeroallergens

Fungi are eukaryotic organisms that grow all over the world, in the presence of moisture and carbohydrates [40,41]. Currently, fungi are organized into eight phyla, three of which produce important aeroallergens: (1) *Zygomycota*; (2) *Ascomycota*; and (3) *Basidiomycota* [41,42,43]. Fungi produce spores on maturity, through both sexual and asexual mechanisms [44,45]. The asexual spores produced by mitosis appear to be the most allergenic [41,46]. In addition to molecules on the surfaces of spores, fungi also secrete enzymes into their environment that can act as allergens in sensitized individuals [41,47]. Fungi spores have low mass and can remain suspended in the air for varying periods of time depending on the weather [48]. Outdoor airborne fungi such as *Cladosporium*, *Alternaria*, *Penicillium*, and *Aspergillus* can trigger allergic responses in sensitized individuals [49]. 

### 2.4. Aeroallergen Measurement and Prediction

Outdoor aeroallergen sampling is largely based on microscopic examination to identify pollen and spores based on morphologic pattern recognition [50,51]. Aeroallergens must be collected and then manually counted, so it is impossible to generally offer real-time data using these methods. Furthermore, it is not yet feasible to provide dense spatial coverage or to offer sub-daily counts on a regular basis, although bi-hourly values are occasionally reported in research [52]. 

Aeroallergen count modeling and forecasting allows for more detailed maps that provide estimates for locations other than sampling sites. This requires knowledge of botanical and meteorological data over several years to assess the variations possible in seasonal pollen characteristics [53]. Land use regression (LUR) modelling is a mapping technique [33] that has been used to explain approximately 79% of the spatial variation in pollen measurements [54]. Results from LUR models are a potential source of relevant information for epidemiologic studies of allergic conditions [33]. Model improvements and their validation by additional local measurements are needed to provide more accurate maps and forecasts for epidemiologic studies and to help allergic individuals and their health care providers reduce the burden of illness [53].

### 2.5. Aeroallergen Impact on Humans

Pollen grains carry noninfectious proteins, so exposure to pollen is an innocuous event for most individuals [55]. For others, however, it triggers an allergic immune response and related symptoms. In recent decades, pollen allergy has grown increasingly prevalent, affecting as much as 40% of the population in regions of Australia, New Zealand, and the United States, and becoming widely recognized as a public health concern [56]. 

Because the primary mode of dispersal for pollen grains and fungi spores is via the air, humans are exposed predominantly through inhalation [57]. The respiratory system is the first point of contact between the human body and the proteins contained in pollen and fungi [48], and that is where inflammation takes place following exposure to allergens [58]. Allergies are diagnosed using ‘skin-prick tests’, which demonstrate the presence of a Type I hypersensitivity, involving IgE, at a site on the skin where a small amount of allergen is introduced through a ‘prick’ [22,59]. For seasonal allergic rhinitis, grass, and tree pollens are the most common allergens [60].

The point at which aeroallergens impact on the respiratory system depends on their size. Aeroallergen size can vary from less than 10 μm to 100 μm, and can be classified into the following groups: very small (<10 μm), small (10–25 μm), medium (26–50 μm), large (51–100 μm), and very large (>100 μm) [61]. According to Szema [62], aeroallergen grains larger than 5 μm deposit in the ocular conjunctiva and nasal mucosa and have the potential to trigger allergic reactions such as conjunctivitis or allergic rhinitis [2,63], and also to asthma exacerbation [64]. Smaller particles than <5 μm, come from allergens such as cat dander, can reach the lungs, and have been linked to asthma onset [65].

### 2.6. Aeroallergens and the Development of Respiratory Allergies

Worldwide, a steep increase in the prevalence of Type I hypersensitivity reactions—such as asthma and allergies—has been documented in every age group [66]. Recent studies report significant differences over such a short timespan that the findings cannot be explained by genetic changes [67,68,69,70]. Better understanding of the environmental factors that affect the development of atopic disorders and their phenotypic expression could lead to better understanding of their increased prevalence. Because pollen grains from anemophilous plants are one of the most important allergen sources in the outdoor air [57], their role in development of asthma and allergy needs further exploration. Spores from fungi are also important aeroallergens that have been linked to allergic reactions [41,46] and in some studies potentially also to the risk of asthma development [71].

Asthma is one of the most severe expressions of an adverse immune response in the respiratory system [72,73]. Moreover, researchers such as de Weger et al. [58] and von Mutius [74] acknowledge the diversity of factors that influence the health impact of aeroallergens and call for better understanding of the extrinsic influences and intrinsic factors that contribute to the new onset of the allergic conditions associated with aeroallergen exposures. Among the possible explanations for the onset of allergic conditions, the hygiene hypothesis has gained widespread acceptance [8]. This hypothesis states that the primary factor underlying increases in autoimmune diseases (including allergic conditions) is the decrease in infectious diseases due to improved hygiene and sanitation, and increased use of vaccines and antibiotics [9]. 

According to Gunawan et al. [23] the allergic potency of aeroallergens is linked to substances that have structural similarity to inflammatory lipid mediators including prostaglandin E_2_ and leukotriene B_4_. Lipid fractions from pollens promote chemotaxis and the activation of neutrophils and eosinophils. Additionally, allergenic pollens release proteases [75]. This proteolytic activity may also be involved in the development of allergic diseases. The combined actions of lipid mediators, proteases, and allergens in the respiratory system promote a disruption of normal cell barriers and a modification of antigen-presenting cells to exhibit predominately Th2 activity. In this microenvironment, the development of incident asthma is more likely [75].

In many children with asthma, the appearance of different allergic conditions follows a sequence that has been labelled the “atopic march” [76]. This sequence connects the different expressions of allergic diseases that vary with age, which often have transient symptoms. Typically, a large exposure to a potential allergen is followed by sensitization, according to the order in which infants are exposed to a predominant allergen: first food allergens (e.g., egg), then indoor allergens (e.g., dust mites), and finally, outdoor allergens (e.g., local anemopilous pollens) [69]. This sequence of exposures and sensitizations generally precedes the sequence of atopic symptoms that begins with food allergy and associated gastrointestinal disorders, continues with atopic dermatitis, and progresses to respiratory allergies [77].

Because the first symptoms of the allergic conditions involved in the “atopic march” sometimes emerge during the first year of life, the first possible window for the environmental exposures responsible for these cases of atopy and asthma is likely to be open during gestation and the first months of life. This window is calculated considering two factors: (1) that only exposures preceding the first symptoms of an illness “can influence its inception” [67] (p. 2231); and (2) that antigens and air components can cross the placental barrier [78].

In other cases, such as pollen-related later-onset symptoms, allergies and asthma appear later in life when individuals are exposed to:

(a) new varieties of pollen through immigration to a new location [79,80] or through the appearance of botanical species in new geographical zones other than their native zones [81];
(b) higher than normal exposures to pollen, including in jobs that require long periods of contact with plants [82]. Occupational allergies and bronchial asthma have been reported in agricultural populations [83,84,85], florists [86], floriculturists [87], carpenters [88], and gardeners [89].

### 2.7. Variability in Sensitivity

A critical question for both pollen and fungi allergy is: how much aeroallergen exposure is needed for an allergic person need to develop sensitization and or exacerbation of existing disease? Most studies of exacerbation have found non-linear responses to indoor and outdoor allergens, indicating a threshold level for response [40,90,91]. In addition to a threshold for clinical symptoms, threshold exposure levels have also been described for the release of biological and inflammatory mediators [92]. The threshold values for fungi spores are generally higher than for pollen grains and range from ≥100 spores/m^3^ for *Alternaria*, to ≥900 spores/m^3^ for *Aspergillus/Penicillium*, to ≥3000 spores/m^3^ for Cladosporium [93,94,95,96]. Although individuals vary widely in their reactivity to allergens, one proposed threshold for outdoor ragweed levels in the United States was estimated at 10 to 20 grains/m^3^ for a period of at least 15 min [40]. During peak ragweed season, concentrations in North America can reach 250 grains/m^3^, and they remain above 100 grains/m^3^ for most of the season [97]. Likewise with tree pollen in Ontario, where mean in-season values of 300 grains/m^3^ were recorded, with peaks reaching even higher [98]. Peaks reported for grass are somewhat lower, between 70 and 110 grains/m^3^ to precipitate symptoms [99]. 

The priming effect is another important phenomenon involved in our understanding of the induction of allergic symptoms. The priming effect describes the transition from a state of minimal symptoms out of season to noticeable/bothersome symptoms after sufficient exposure to an allergen. It is formally defined as an increase in reactivity of the nasal membrane following repeated exposure [100]. Individuals who are already experiencing symptoms due to a perennial allergen (dust, pets, etc.) [101] or to high levels of air pollution [64] are more sensitive to subsequent exposure to aeroallergens. Conversely, when individuals with multiple sensitizations are ‘primed’ by an ongoing pollen season, they react more readily to allergens found in the home environment, such as dust [10,101]. The priming effect is relevant to consider when developing pollen warning or forecasting system in conjunction with existing systems for outdoor air pollutants. It demonstrates that those who suffer from seasonal allergies may have the ability to initiate short-term treatment such as antihistamines or sublingual immunotherapy within the first 3–5 days of the season to block the development of severe symptoms. However, those who are already suffering from poorly controlled allergic rhinitis or asthma are in danger of reacting severely and almost immediately upon the arrival of pollen season due to pre-priming [102].

### 2.8. Development of Respiratory Allergies: Risk/Protective Factors

The most promising mechanism that can explain the development of allergies involves interactions between individual genetic susceptibility and environmental exposures [103]. For example, gene–environment interactions have been demonstrated with changes in microRNA [3] and bronchial epithelial DNA methylation, after exposures to diesel exhaust [104]. For individuals with atopy-prone or immune modulated genotypes, environmental exposures play a role in the onset of an allergic disorder [105], including exposures to aeroallergens [2,106]. However, the environmental effect may be either detrimental or protective; Tovey et al. [91] found a non-linear relationship between mite allergen exposure and sensitization and asthma, with children on the lowest and highest quintile of exposure less likely to have both sensitization and asthma compared with the middle range of exposures. 

### 2.9. Protective Factors

An environmental exposure can be considered as a protective factor if it is linked to decreased risk of the development of a specific condition [105].

#### 2.9.1. Animals

An increasing number of studies indicate that exposure to domestic animals (pets or livestock) in early life decreases the risk of asthma [67], aeroallergen sensitization [107], and respiratory allergic disease as a whole [108]. The immune system modulation caused by these exposures is aligned with the hygiene hypothesis in which microbial exposures shift the immune response towards Th1 predominance [8,109,110].

Early and prolonged contact with animals [111] seems to have a protective effect against sensitization to aeroallergens, particularly if the exposure happens during the two first years of life [107]. The benefit appears to continue throughout the life course [112]. Exposures to farm animals seem to reduce specific aeroallergen IgE antibody production [113], particularly with higher exposures [114].

#### 2.9.2. Breastfeeding

Breastfeeding, defined as the consumption of human breast milk through infant suckling directly from the breast, is acknowledged as an optimal source of infant nutrition [115]. Despite the short-term clinical and societal benefits, evidence has been mixed on the role of breastfeeding in the development of atopic conditions. 

Breastfeeding has been associated with decreased risk of child atopy and the different phenotypes of asthma [116]. Although some factors such as maternal atopy might confound this association [117], reduced risk is present mostly with exclusive breastfeeding when compared with consumption of expressed milk or combined breast milk and formula [115]. Risk reduction extends well into school age, but is most pronounced in children 0–2 years [116].

### 2.10. Risk Factors

To consider an environmental element as a risk factor, it is necessary to demonstrate a positive exposure–response relationship with the allergic outcome of interest [103]. Interactions between pollen and the following environmental conditions have been studied and linked to increased risk for the development of an allergic condition [118]. 

#### 2.10.1. Weather

In northern climates such as Canada, pollen production, dispersal, and airborne lifespan are highly dependent on the weather. In general, trees bloom in the early spring, grasses bloom late spring to early summer, and weeds pollinate in the fall [40]. Ragweed is in late August until the first frost, and thus ragweed season can be cut short by an early frost [32,119]. The weather in the current year has less effect on the intensity of the tree pollen season than the weather in the previous year, because trees produce pollen in the summer and fall, then release it when a sufficient number of warm days occur in the following spring [32,119]. Thus, the amount of tree pollen released depends on the weather in the previous year, but the timing of release depends on the current spring weather [32]. 

Diurnal changes in pollen levels have also been observed. As ragweed pollen is released in the morning, peak concentrations are reached around noon in the absence of high winds or rain [120]. Depending on the temperature, humidity, and wind conditions, the counts may drop at night or remain elevated [40,121]. Mature pollen grains tend to be released when the relative humidity drops, and they remain airborne longer at low humidity, low wind speeds, and high atmospheric pressure [120]. Meteorological conditions also affect fungi sporulation, as many grow well when conditions are wet and they produce spores as a survival mechanism when conditions are dry [40,121]. 

Precipitation can temporarily clear particles and aeroallergens from the air, although some pollen grains or fungi spores may be resuspended after a brief rainfall [36,122]. Exposure to water may also increase the formation of both pollen and fungi aeroallergens in a 24-h timeframe [40]. Considering the added complexity of rainfall, it is possible that the presence of high counts may be sufficient, but not necessary, for significant aeroallergen exposure to occur, due to the ruptured aeroallergen grains that are not accurately counted during these events [40].

Thunderstorms can be associated with asthma exacerbations, with steep increases in the use of emergency services and increased mortality [123,124,125]. Thunderstorms during pollen season are known to carry whole and ruptured pollen grains at the ground level where wind outflows distribute them with larger geographic coverage than under normal conditions [126]. Climate change has been linked to an increase in frequency of thunderstorms in some areas [127].

#### 2.10.2. Seasonality

The division of the year into four seasons (spring, summer, fall, and winter) takes into consideration variables that undergo yearly cyclical changes, such as day length, temperature, and humidity. The influence of these factors on aeroallergens can be direct and indirect [128], and all have been associated with allergic respiratory morbidity [129,130,131] and mortality [132,133]. There is some indication of an association between season of birth on the development of asthma and allergies [134]. If an individual is exposed to high aeroallergen counts in utero and/or during the sensitive immune system development period of the first months of life, there may be at risk of atopic disease later in life [135,136,137].

#### 2.10.3. Urbanization

Living in urban areas, as opposed to rural and semi-rural areas, is a known risk factor for the development of aeroallergen-induced respiratory allergy [128,138]. There is evidence that highly allergenic species of fungi are present at higher levels in the outdoor air in cities compared with rural environments [139]. Differences between aeroallergen counts (higher in the rural areas) and species diversity (lower in urban areas) may drive some of the differences between rural and urban influence on the onset of allergies and asthma [140]. Additionally, the higher levels of air pollutants predominant in urban areas can interact with airborne pollen grains, which is likely to exacerbate allergic conditions and respiratory distress [128]. Two thirds of the global population will live in urban areas by 2020, and an increased risk for aeroallergen-induced respiratory allergy is expected [127].

#### 2.10.4. Air Pollution

The interactions between air pollution and aeroallergens are complex and occur both in the atmosphere and in the airways [141]. Ziska et al. [142] have demonstrated that outdoor air pollution can prompt an increase in pollen production by certain herbaceous species, similar to the effects seen with increased CO_2_. A number of studies have also shown that air pollution may increase the allergenicity of pollen and/or fungi, because air pollutants can attach to the surface of pollen grains and potentially alter their allergenic potential and morphology by making the surface of the pollen grain coating more fragile [128,143,144]. Timing of exposure is important, and co-occurence of pollen/spore release and increased air pollution episodes appears to be the most harmful in terms of increased allergenicity. Ragweed pollen is released from early to mid-morning, peaking around noon [145]. Air pollution monitoring data from various cities has shown that traffic-related air pollution (TRAP) follows a diurnal pattern with an increase during the daily rush hours [146,147,148]. Air pollution in combination with sunlight can lead to the photochemical production of ground-level ozone, which typically peaks in the afternoon. Thus, the diurnal patterns of pollen release, TRAP, and ozone concentrations in cities may place high levels of aeroallergens in contact with relatively high daily levels of air pollution, potentially leading to ambient modifications of the allergenicity of aeroallergens [146].

In the airway, oxidative stress has been identified as a major biologic pathway for the effects of most of the air pollutants components (e.g., PM_2.5_, ground-level ozone, nitrogen dioxide (NO_2_) and sulfur dioxide (SO_2_) [149,150]. Air pollutants allow for easier penetration of pollen and spore allergens because they trigger damage to the airway mucociliary clearance mechanisms [128,141]. Particulate matter smaller than 2.5 μm (PM_2.5_) reaches deeply in the lungs and acts as an adjuvant that increases production of IgE [3,151,152]. Furthermore, exposure to diesel exhaust is known to impact DNA methylation and impaired regulatory T-cell functions, two mechanisms relevant for the immune response [104]. Changes in FEV1 following allergen exposure have also been modified by controlled diesel exhaust exposure and *GST* genotype in a gene–environment interaction manner [152]. These GST variants are commonly present in the general population (i.e., from 23% to 62% depending on the ethnic group, in the USA) [153], so many people might experience amplified effects of air pollution–aeroallergen co-exposures in the outdoor environment.

### 2.11. Fundamentals of the Allergic Response

**Figure 3:**
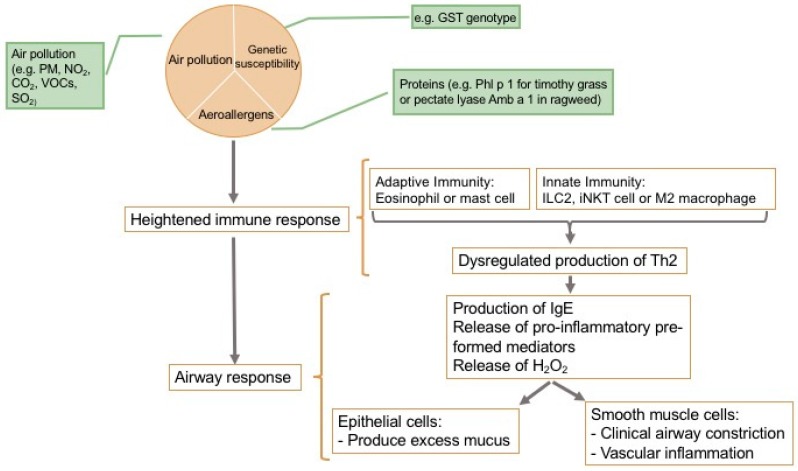
Adverse immune response in the airway, adapted from [158].

In humans, immune responses can range from diminished (i.e., immunosuppression) to heightened (i.e., allergies) [72], with a biologically appropriate response in the middle of these extremes (i.e., appropriate recognition and response to harmful pathogens) [55]. Pollen grains carry cytoplasmic granules that release a number of proteins and glycoproteins when they are exposed to water or airborne pollutants. These proteins instigate the adverse immune response in sensitized individuals (Figure 3) [128,154,155,156]. In addition, single and repeated exposures to pollen in allergic individuals have been shown to induce epigenetic changes, both in the blood and the nasal tissue [157].

The impact of aeroallergens has been documented in every section of the airway. The nose is the first point of contact with aeroallergens, and the histamine released due to interaction with aeroallergens increases tissue swelling and permeability, causing rhinitis and rhinorrhea [59]. Rhinitis is a risk factor for the development of asthma [60] and allergic rhinitis is most commonly due to pollen allergy [159].

Due to the many similarities between the nasal and bronchial mucosa and their reactions to aeroallergens, the “one airway, one disease” concept has been proposed for allergic rhinitis and allergic asthma [160]. Approximately 80% of patients with allergic asthma also have allergic rhinitis symptoms upon exposure to aeroallergens, but not all people with allergic rhinitis experience asthma symptoms [160,161,162]. Sensitization to aeroallergens is a major contributing factor to this discrepancy [163].

The hyper-responsiveness of the airways and subsequent constriction following allergen exposure are distinctive clinical findings in asthma [103,164,165]. Constriction is due to inflammatory processes and airway remodeling. Reversibility of the inflammation over time or by medication are also considered in asthma diagnosis [74]. Wheeze, cough, and paroxysmal dyspnea are less specific clinical signs of asthma. Persistent symptoms, triggered by the Th2 and IgE interactions, lead to structural changes and remodeling of the airway [166].

Asthma is a complex disease that can be understood as a syndrome, because different pathways can result in diverse phenotypes [74]. Several clinical phenotypes of asthma display differences in severity, inflammatory pattern, and comorbidity with allergic diseases (since not all asthma is atopic) [167]. Reversible bronchoconstriction, either spontaneously or after bronchodilator treatment, remains a common process in all the phenotypes [166]. 

Among respiratory conditions, asthma is one of the major non-communicable chronic diseases. Globally, approximately 235 million people currently suffer from asthma [168]. In high-income countries such as Canada, prevalence rose for many years from the early 1960s to 2000 [19,128] and recently seems to have reached a plateau [126,169].

### 2.12. Prevalence of Respiratory Allergies in Canada

**Figure 4:**
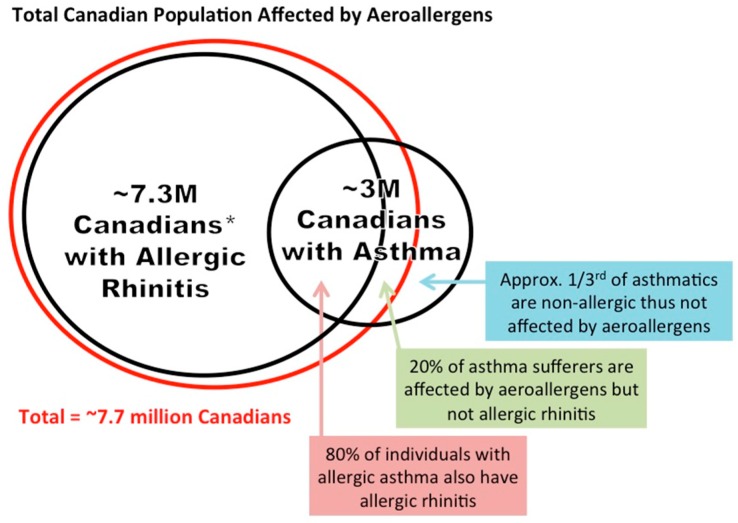
Total Canadian population affected by aeroallergens. This estimation of the total allergic population is based on the conservative estimate that 20% of the Canadian population is affected by allergic rhinitis, and the total population of Canada used in this calculation was based on 2016 data from Statistics Canada [175].

Allergic rhinitis is highly prevalent in Canada, affecting approximately 20–25% of the population [170]. Asthma is estimated to affect about 3 million Canadians, and between 12% [171] to 25% of Canadian children [172,173,174]. Approximately two-thirds of people with asthma are allergic to aeroallergens, and these allergens act as triggers for asthma exacerbations [15]. Overall, approximately 7.7 million were people affected by aeroallergens in Canada in 2016 (Figure 4) [175]. High concentrations of ambient aeroallergens, including tree pollen, fungal spores, and others, have been associated with increased risk of early delivery [176], myocardial infarction [98], as well as with asthma-related emergency department visits and hospitalizations in cities across Canada, demonstrating that severe symptoms are related to aeroallergen counts across the country [177,178,179,180,181]. 

### 2.13. Economic Costs Associated with Respiratory Allergies

Atopic conditions represent a large burden on the economy. A study in British Columbia found that excess costs to the healthcare system in patients with asthma were $1028.00 per patient/year, compared with a control group [182]. This included the direct costs of asthma, as well as publicly-funded healthcare costs related to comorbid conditions in people with asthma, including allergic rhinitis [182]. Asthma is also the leading driver of health care costs for children in Canada, estimated at over $2 billion per year [183]. Recent estimates suggest that in a single year (between 2014–2015), asthma attacks triggered over 70,000 emergency room visits [184]. The costs of asthma to the Canadian economy are also expected to rise to almost $4 billion per year by 2030, more than double the current cost [185].

### 2.14. Effects of Climate Change on Aeroallergens

**Figure 5:**
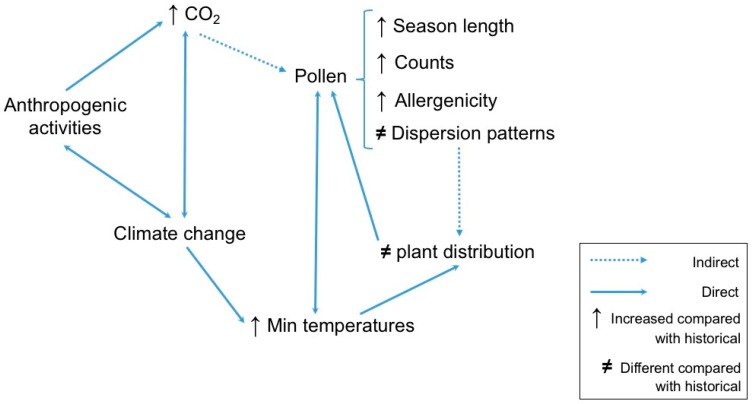
Effects of climate change on aeroallergens.

Levels of some specific aeroallergens are on the rise in particular regions, and some of this increase has been linked to anthropogenic climate change [186]. Anthropogenic climate change has been documented over the past 100 years and is projected to continue to intensify [81], driven in part by increases in temperature [138,187]. As seen on Figure 5, the increasing planetary temperatures affect plant biology through the timing and length of the growing seasons [188], increased production and allergenicity of pollen, and shifts in range of species [186,189].

Due to the warmer temperatures, pollen seasons will start earlier and end later [66,138,155,190]. Warming by latitude has been associated with increased length of the ragweed pollen season in North America, increasing by 27 days between 1995 and 2009 [149]. Earlier seasonal starts have also been found for *Juniperus*, *Ulmus*, and *Morus* [81]. In Western Europe, spring events have advanced by six days [191] and changes in birch pollen season [192] and *Artemisia* have been described [193]. Changes in length will lead to changes in human exposure [194] and potentially in sensitization [2,189].

Increased pollen counts have been associated with the current levels of CO_2_, when compared with CO_2_ levels of the previous century [195] or even between different decades (2004 and 2013 in Ariano et al.) [186,196]. Increases in the atmospheric concentrations of CO_2_ impact the reproductive processes in plants, leading them to produce more pollen [111,197,198] with significantly stronger allergenicity has been found in pollen [81,193,199]. This increased allergenicity is due to more diversity in the heterogeneity of the antigenic proteins in pollen grains [62]. 

Climate change affects many meteorological variables that participate in the pollen dispersal and deposition (i.e., humidity, precipitation, and temperature) [127]. Changes in dispersion, both region- and species-specific, can potentially expose (and sensitize) populations to novel allergens [141]. These changes in dispersion will also impact plant distribution, as species that could not survive in previously hostile environments can potentially thrive because of changes in temperature and precipitation [193]. The proliferation of anemophilous plants where those species were not previously prevalent exposes atopy-prone individuals to new pollens [200].

### 2.15. Environment and Health Interventions for Patients with Respiratory Allergies

Allergic asthma is managed through allergen avoidance and pharmacotherapy to achieve and maintain asthma control [201].

The Canadian guidelines state that, after their diagnoses, patients and/or caregivers should receive a written asthma action plan and self-management education [201]. Pharmacotherapy should be part of the written asthma plan and is designed to be long-term to prevent exacerbations. Thus, aside from the self-administration of short-acting beta-agonists as needed, pharmacotherapy for asthma is not typically altered based on current aeroallergen levels except to ensure that such long-term controller medications are used properly and as prescribed. For those who are allergic to seasonal aeroallergens, knowledge of current or upcoming pollen or fungi counts may aid in both avoidance and the optimization of pharmacotherapy [127]. If certain aeroallergens are known to exacerbate individual asthma symptoms, allergen avoidance is one option (although it may not always be feasible), and immunotherapy may be recommended in advance of the allergy season. Most people with allergic asthma also have allergic rhinitis, and the treatment of both disorders during times of increased aeroallergen presence has been shown to be critical to the maintenance of asthma control; therefore, supporting the optimization of allergic rhinitis therapy by providing information on aeroallergen levels may have a profound impact on many patients with allergic asthma [162]. 

The efficacies of most prescribed medications for allergic rhinitis are enhanced if they are taken consistently and/or before symptom onset [170]. Thus, reminders and warnings that various aeroallergen seasons, wildfire exposures, elevated air pollution episodes or thunderstorms are approaching may allow Canadians to ensure they have visited a healthcare provider, refilled prescriptions, and begun taking preventative medications according to their management plan [202]. 

## 3. Conclusions

Pollen and fungi are important allergen in the outdoor air [57]. Aeroallergens play a critical role in the development of allergic conditions through sensitization, particularly in those individuals with genetic predisposition [203]. 

Pollen and fungi do not act in isolation. Other environmental exposures (e.g., TRAP) also play a role in the interaction between air and respiratory tissue [103]. Environmental exposures can act as risk or protective factors in the development of allergy to aeroallergens [204]. After the sensitization takes place and the affected individual develops symptoms, further exposures to risk factors may cause symptom exacerbations that will diminish with reduced exposure or with medical treatment [67]. 

An aeroallergen alert system could be used to communicate with sensitized individuals and advise them to control their exposure when aeroallergen counts are high. An aeroallergen alert system would benefit from a model of aeroallergen production and dispersion that also incorporates weather-related variations and long-term impacts of climate change and could be coupled with existing air quality predictions. Given the documented clinical cross-reactivity between pollens from similar species [30], the idea of reporting total tree, grass, and ragweed pollens is better suited to the general public for simplicity and cost minimization, while counts of individual species would still have value for modeling and research.

Climate change has the potential to increase the length of aeroallergen seasons as well as pollen counts, geographical coverage, and allergenicity [19]. These increases will also increase human exposure to the allergenic proteins found in pollen grains [155,193]. Future increases in the incidence and prevalence of respiratory allergies and asthma are predicted [194], accompanied by an increase in health care expenses allotted to the care of these conditions [62]. Longitudinal studies are needed to examine long-term trends beyond the yearly variations due to variation in aeroallergen seasons. Such studies would help explain regional differences in risk and increase our understanding of the impact of climate change on pollens and fungi [18].

Better understanding of both pollen and fungi counts, phenology, geographic coverage, and interaction with air pollutants is needed to provide the information that atopic individuals could use to modify their environmental exposures [127], and also to shape government initiatives such as alert systems and landscaping in urban zones [205]. 

## Supplementary Materials

The following is available online at http://www.mdpi.com/1660-4601/15/8/1577/s1, Table S1: Plant-Derived Aeroallergens in the Major Floristic Zones of Canada.
