# Supplementary material for: Aeroallergens in Canada: Distribution, Public Health Impacts, and Opportunities for Prevention
Source: Int J Environ Res Public Health. 2018 Jul 25;15(8):1577. doi: 10.3390/ijerph15081577 (PMC6121311; doi:10.3390/ijerph15081577)
Supplement: Supplementary file 1 [file ijerph-15-01577-s001.pdf]

Table S1. Plant-Derived Aeroallergens in the Major Floristic Zones of Canada

Floristic Zone	Trees		Grasses and Weeds	
	**Common Name**	**Genus**	**Common Name**	**Genus**
Northwest Coastal: Southwestern British Columbia (BC)	Cedar	*Juniperus*	Rye & Orchard grass	*Lolium & Dactylis*
	Fir	*Abies*	Fescue grass	*Festuca*
	Pine	*Pinus*	Brome/chess grass	*Bromus*
	Alder	*Alnus*	Timothy & velvet grass	*Phleum & Holcus*
	Birch	*Betula*	Sweet grass	*Anthoxanthum*
	Hazel	*Corylus*	Plantain & Amaranths	*Plantago & Amaranthus*
	Oak	*Quercus*	Short ragweed	*Ambrosia*
	Cypress	*Cupressaceae*	Chenopods & Orach	*Kochia & Atriplex*
			Dock & Sorrel	*Rumex*
Northern Forest : Yukon, NWT, Nunavut, Newfoundland and Labrador, Northern BC, Alberta, Manitoba, Ontario & Quebec	Juniper	*Juniperus*	Mugwort	*Artemisia*
	Cedar & Birch	*Cedrus& Betula*	Nettle	*Urtica*
	Pine & Fir	*Pinus & Abies*	Chenopods	*Chenopdium & Kochia*
	Alder & Aspen	*Alnus & Populus*	Pigweeds	*Amaranthus*
	Willow & Maple	*Salix & Acer*	Pasture grasses (*low*)	
Rocky Mountain: Southeastern BC, Southern Alberta	Mountain cedar	*Juniperus*	Rye & Orchard grass	*Lolium & Dactylis*
	Pine	*Pinus*	Fescue grass	*Festuca*
	Aspen/Cottonwood	*Populus*	Timothy & Chess grass	*Bromus & Phleum*
	Willow	*Salix*	Tumbleweeds	*Kochia & Salsol*
	Maple/Boxelder	*Acer*	Pigweed	*Amaranthus*
	Oak	*Quercus*	Dock & Sage	*Rumex & Artemisia*
	Ash	*Fraxinus*	Giant/western ragweed	*Ambrosia*
Central Plains: Southern Alberta, Saskatchewan and Manitoba	Red cedar	*Juniperus*	Rye & Orchard grass	*Lolium & Dactylis*
	Pine & Beech	*Pinus & Fagus*	Fescue grass	*Festuca*
	Red/white oak	*Quercus*	Timothy & chess grass	*Phleum & Bromus*
	Elm & Ash	*Ulmus & Fraxinus*	Sweet grass	*Anthoxanthum*
	Maple/Box elder	*Acer*	Short/giant ragweed	*Ambrosia*
	Alder & Birch	*Alnus & Betula*	Mugwort	*Artemisia*
	Walnut & Pecan	*Juglans*	Pigweed	*Amaranthus*
	Locust &Hawthorn	*Robinia &Crategus*	Lamb's quarter	*Chenopodium*
	Cottonwood	*Populus*	Nettle & Dock/sorrel	*Urtica & Rumex*
	Willow & Locust	*Salix & Robina*	Plantain & Chenopods	*Plantago & Kochia*
	Sycamore&Hickory	*Plantanus & Carya*	Pigweed & Waterhemp	*Amaranthus & Ancida*
Eastern Agricultural: Southern Ontario, Southern Quebec, New Brunswick, Nova Scotia, Prince Edward Island	Cedar & Pine	*Juniperus & Pinus*	Rye & Orchard grass	*Lolium & Dactylis*
	Oak & Beech	*Quercus & Fagus*	Fescue & chess grass	*Festuca & Bromus*
	Elm & Ash	*Ulmus & Fraxinus*	Timothy & Sweet grass	*Phleum&Anthoxanthum*
	Sycamore	*Plantanus*	Short/giant ragweed	*Ambrosia*
	Maple & Cypress	*Acer&Cupressaceae*	Mugwort & Pigweed	*Artemisia&Amaranthus*
	Alder & Birch	*Alnus & Betula*	Lamb's quarter	*Chenopodium*
	Hickory & Locust	*Carya & Robinia*	Dock & sorrel	*Rumex*
	Hawthorn	*Crategus*	Plantain & Nettle	*Plantago & Urtica*
	Walnut & Pecan	*Juglans*		

Adapted from [14].
